# Supplementary material for: An exploratory survey about using ChatGPT in education, healthcare, and research
Source: PLoS One. 2023 Oct 5;18(10):e0292216. doi: 10.1371/journal.pone.0292216 (PMC10553335; doi:10.1371/journal.pone.0292216)
Supplement: S1 File — Full survey question and response options as administered to the audience. (PDF) [file pone.0292216.s001.pdf]

S1 File. Survey delivered via Slido.

1. **What's your current role?**
    - Medical Student, Resident, Fellow
    - Graduate Student, Postdoc Researcher
    - Clinical Faculty
    - Research Faculty
    - Administrative Staff
    - Other
2. **Have you used ChatGPT?**
    - Yes
    - No
3. **How interested are you in using ChatGPT in your day to day work?**
    - To a Great Extent
    - Somewhat
    - Very Little
    - Not at All
4. **Can ChatGPT be used in education?**
    - No, it should be banned
    - Yes, it should be actively incorporated
    - I don't know, it is too early to make a statement
5. **Can ChatGPT be used for science?**
    - No, it should not be used at all
    - Yes, but it should only be used to help brainstorm
    - Yes, as long as its use is transparently disclosed
    - Yes, disclosure is NOT needed
    - I don't know, it is too early to make a statement
6. **Can ChatGPT be used in healthcare?**
    - No, it should not be used at all
    - Yes, it can only be used to help write administrative content such as emails to insurance companies or to patients
    - Yes, it can be used for any purpose
    - I don't know, it is too early to make a statement
7. **Using one keyword, describe challenges of using ChatGPT**
8. **Using one keyword, describe benefits of using ChatGPT**
