# Supplementary material for: An exploratory survey about using ChatGPT in education, healthcare, and research
Source: PLoS One. 2023 Oct 5;18(10):e0292216. doi: 10.1371/journal.pone.0292216 (PMC10553335; doi:10.1371/journal.pone.0292216)
Supplement: Attachment — Submitted filename: Response to reviewer comments.docx [file pone.0292216.s002.docx]

Dear Dr. Clark and the *PLOS One* editorial team,

Thank you for your comments. We have revised our manuscript and included a point-by-point response to the reviewer comments below. In response to other comments raised:

Formatting: We have reformatted our document per *PLOS ONE*’s style requirements as requested. Our supplemental file includes a table and a list of questions both with relevant captions. Please let us know if there are additional formatting changes to be made.

Data sharing: We have published our dataset on Zenodo at doi 10.5281/zenodo.7789186 and all code needed to completely reproduce our results at Github repository <https://github.com/cloverbunny/gptsurvey/blob/main/gptsurvey.ipynb>. This is articulated in our Data and Code Availability statements.

Regarding Word Cloud Figure licensing: This is not a copyrighted image, as we generated this with our specific data. We contacted the author of the website to clarify and request his signature on the provided document; he said it was not necessary for us to obtain formal documentation of use from him, see screenshot of email communication and disclaimer below. The word cloud generator website explicitly contains the permission: “The generated word clouds may be used for any purpose.”

In the following pages of this document, you can see our responses to reviewer comments.

Reviewer #1 Comment #1: Thank you for the opportunity to review this manuscript on ChatGPT and similar AI tools. As an academic, the topic holds great relevance and has been widely discussed. However, I believe that a comprehensive review of existing literature is essential for advancing knowledge in any field. Literature reviews serve as the foundation for new research, identifying gaps, building upon previous studies, and contributing to the overall body of knowledge. Unfortunately, this manuscript suggests that the current literature is insufficient in providing a comprehensive understanding of the subject matter.
In some cases, the insufficiency of literature can be attributed to the field being relatively new or rapidly evolving. New developments and paradigms take time to be reflected in the literature, resulting in limited studies, replication, and consensus on key concepts or theories. This dynamic nature poses challenges for researchers attempting to establish a comprehensive literature base. Recognizing the insufficiency of existing literature is crucial for addressing gaps and advancing knowledge in the field. However, this manuscript falls short in providing a full and comprehensive picture of how ChatGPT can be utilized in broader contexts, such as education, healthcare, and research. The literature on these specific applications was limited to one paragraph each, which is insufficient.

Reviewer #1 Response #1: We thank the reviewer for the comments and agree that this is a rapidly developing field. We have expanded upon our introduction and background in all three realms of education, healthcare, and research. We have furthermore linked to more comprehensive reviews for interested readers for detailed discussion and literature review beyond the scope of our paper.

Reviewer #1 Comment #2: Additionally, the manuscript heavily relies on unnecessary graphics and images, which could have been condensed to reduce fluff. The demographic information could have been presented more concisely. Furthermore, the discussion section lacks a clear data interpretation process and a deeper understanding of the results. It appears that the researchers randomly selected comments without providing a comprehensive analysis.

Reviewer #1 Response #2: We have removed and condensed some of our figures and presented our demographic information in Table 1 in a concise format. We included all comments raised during the Question and Answer session without any random selection. We have expanded upon our Discussion.

Reviewer #1 Comment #3: Moreover, there were frequent APA errors throughout the manuscript, which need to be addressed.

Reviewer #1 Response #3: PLOS One waives all formatting requirements for initial submissions, which is why we did not initially adhere strictly to these guidelines. We have made stylistic revisions in this new document and adhered to the style as outlined by PLOS. We are happy to work on any additional formatting requests from a copy editor.

Reviewer #2 Comments: The paper is not as per journal format.
The font sizes are different in several placesInstead of bibliography it should be references If possible Need to cite more papers related to chatGPT and related to this research
Figures titles are not as per journal standards and make sure to use any templates related to plus one available

Reviewer #2 Responses: We have corrected these stylistic errors, cited more papers related to the research, and corrected figure titles and references. PLOS One waives all formatting requirements for initial submissions, which is why we did not initially adhere strictly to these guidelines. We are happy to consider additional formatting changes as suggested by a copy editor.

 Dear Dr. Hosseini,

Thank you for submitting your manuscript to PLOS ONE. After careful consideration, we feel that it has merit but does not fully meet PLOS ONE’s publication criteria as it currently stands. Therefore, we invite you to submit a revised version of the manuscript that addresses the points raised during the review process.

Please submit your revised manuscript by Jul 29 2023 11:59PM. If you will need more time than this to complete your revisions, please reply to this message or contact the journal office at [plosone@plos.org](mailto:plosone@plos.org). When you're ready to submit your revision, log on to https://www.editorialmanager.com/pone/ and select the 'Submissions Needing Revision' folder to locate your manuscript file.

The reviewers and I find that the manuscript can contribute to the field but feel that it needs more work on the literature review as well as in the discussion.  Please see comments that are included.

Please include the following items when submitting your revised manuscript:

- A rebuttal letter that responds to each point raised by the academic editor and reviewer(s). You should upload this letter as a separate file labeled 'Response to Reviewers'.
- A marked-up copy of your manuscript that highlights changes made to the original version. You should upload this as a separate file labeled 'Revised Manuscript with Track Changes'.
- An unmarked version of your revised paper without tracked changes. You should upload this as a separate file labeled 'Manuscript'.

If you would like to make changes to your financial disclosure, please include your updated statement in your cover letter. Guidelines for resubmitting your figure files are available below the reviewer comments at the end of this letter.

If applicable, we recommend that you deposit your laboratory protocols in protocols.io to enhance the reproducibility of your results. Protocols.io assigns your protocol its own identifier (DOI) so that it can be cited independently in the future. For instructions see: <https://journals.plos.org/plosone/s/submission-guidelines#loc-laboratory-protocols>. Additionally, PLOS ONE offers an option for publishing peer-reviewed Lab Protocol articles, which describe protocols hosted on protocols.io. Read more information on sharing protocols at <https://plos.org/protocols?utm_medium=editorial-email&utm_source=authorletters&utm_campaign=protocols>.

We look forward to receiving your revised manuscript.

Kind regards,

Mary Diane Clark, PhD
Academic Editor
PLOS ONE

Journal requirements:

When submitting your revision, we need you to address these additional requirements.

1. Please ensure that your manuscript meets PLOS ONE's style requirements, including those for file naming. The PLOS ONE style templates can be found at

https://journals.plos.org/plosone/s/file?id=wjVg/PLOSOne_formatting_sample_main_body.pdf and

https://journals.plos.org/plosone/s/file?id=ba62/PLOSOne_formatting_sample_title_authors_affiliations.pdf

2. We note that you have indicated that data from this study are available upon request. PLOS only allows data to be available upon request if there are legal or ethical restrictions on sharing data publicly. For more information on unacceptable data access restrictions, please see http://journals.plos.org/plosone/s/data-availability#loc-unacceptable-data-access-restrictions.

In your revised cover letter, please address the following prompts:

a) If there are ethical or legal restrictions on sharing a de-identified data set, please explain them in detail (e.g., data contain potentially sensitive information, data are owned by a third-party organization, etc.) and who has imposed them (e.g., an ethics committee). Please also provide contact information for a data access committee, ethics committee, or other institutional body to which data requests may be sent.

We have published our dataset on Zenodo at doi 10.5281/zenodo.7789186 and all code needed to completely reproduce our results at Github repository <https://github.com/cloverbunny/gptsurvey/blob/main/gptsurvey.ipynb>. This is articulated in our Data and Code Availability statements.

b) If there are no restrictions, please upload the minimal anonymized data set necessary to replicate your study findings as either Supporting Information files or to a stable, public repository and provide us with the relevant URLs, DOIs, or accession numbers. For a list of acceptable repositories, please see <http://journals.plos.org/plosone/s/data-availability#loc-recommended-repositories>.

See above.

We will update your Data Availability statement on your behalf to reflect the information you provide.

3. Please include captions for your Supporting Information files at the end of your manuscript, and update any in-text citations to match accordingly. Please see our Supporting Information guidelines for more information: <http://journals.plos.org/plosone/s/supporting-information>.

Our supplemental file includes a table and a list of questions both with relevant captions.

4. We note that Figure 7 in your submission contain copyrighted images. All PLOS content is published under the Creative Commons Attribution License (CC BY 4.0), which means that the manuscript, images, and Supporting Information files will be freely available online, and any third party is permitted to access, download, copy, distribute, and use these materials in any way, even commercially, with proper attribution. For more information, see our copyright guidelines: <http://journals.plos.org/plosone/s/licenses-and-copyright>.

We have generated word clouds using a free service and mention this in the text. The word cloud generator notes in their disclaimer that “The generated word clouds may be used for any purpose.” We contacted the author of the website who said it was not necessary for us to obtain formal documentation of use from him, see screenshot of email communication and disclaimer below.

We require you to either (1) present written permission from the copyright holder to publish these figures specifically under the CC BY 4.0 license, or (2) remove the figures from your submission:

a. You may seek permission from the original copyright holder of Figure 7 to publish the content specifically under the CC BY 4.0 license.

We recommend that you contact the original copyright holder with the Content Permission Form (http://journals.plos.org/plosone/s/file?id=7c09/content-permission-form.pdf) and the following text:

“I request permission for the open-access journal PLOS ONE to publish XXX under the Creative Commons Attribution License (CCAL) CC BY 4.0 (http://creativecommons.org/licenses/by/4.0/). Please be aware that this license allows unrestricted use and distribution, even commercially, by third parties. Please reply and provide explicit written permission to publish XXX under a CC BY license and complete the attached form.”

Please upload the completed Content Permission Form or other proof of granted permissions as an "Other" file with your submission.

In the figure caption of the copyrighted figure, please include the following text: “Reprinted from [ref] under a CC BY license, with permission from [name of publisher], original copyright [original copyright year].”

b.If you are unable to obtain permission from the original copyright holder to publish these figures under the CC BY 4.0 license or if the copyright holder’s requirements are incompatible with the CC BY 4.0 license, please either i) remove the figure or ii) supply a replacement figure that complies with the CC BY 4.0 license. Please check copyright information on all replacement figures and update the figure caption with source information. If applicable, please specify in the figure caption text when a figure is similar but not identical to the original image and is therefore for illustrative purposes only.

Additional Editor Comments:

Thank you for submitting the article to PLOS ONE, the topic is interesting and important. The reviewers provide guidance for how they feel that the manuscript needs to be edited. Please pay attention to their comments and make all of their suggested changes. I have concerns about your discussion as it feels more like a summary of your results than an interpretation of your results. As noted by San Jose State University Writing Center (http://www.sjsu.edu/writingcenter) the discussion should include three necessary steps: interpretation, analysis, and explanation. Why are your results important and where do they fit in with what we already know. The questions a discussion should address include how or why the use of Chat GPT is helpful or harmful. What is the meaning of your findings.

We look forward to your revised manuscript


[Note: HTML markup is below. Please do not edit.]

Reviewers' comments:

Reviewer's Responses to Questions

**Comments to the Author**

1. Is the manuscript technically sound, and do the data support the conclusions?

The manuscript must describe a technically sound piece of scientific research with data that supports the conclusions. Experiments must have been conducted rigorously, with appropriate controls, replication, and sample sizes. The conclusions must be drawn appropriately based on the data presented.

Reviewer #1: Partly

Reviewer #2: Yes

2. Has the statistical analysis been performed appropriately and rigorously?

Reviewer #1: Yes

Reviewer #2: Yes

3. Have the authors made all data underlying the findings in their manuscript fully available?

The [PLOS Data policy](http://www.plosone.org/static/policies.action#sharing) requires authors to make all data underlying the findings described in their manuscript fully available without restriction, with rare exception (please refer to the Data Availability Statement in the manuscript PDF file). The data should be provided as part of the manuscript or its supporting information, or deposited to a public repository. For example, in addition to summary statistics, the data points behind means, medians and variance measures should be available. If there are restrictions on publicly sharing data—e.g. participant privacy or use of data from a third party—those must be specified.

Reviewer #1: Yes

Reviewer #2: Yes

4. Is the manuscript presented in an intelligible fashion and written in standard English?

PLOS ONE does not copyedit accepted manuscripts, so the language in submitted articles must be clear, correct, and unambiguous. Any typographical or grammatical errors should be corrected at revision, so please note any specific errors here.

Reviewer #1: Yes

Reviewer #2: Yes

5. Review Comments to the Author

Please use the space provided to explain your answers to the questions above. You may also include additional comments for the author, including concerns about dual publication, research ethics, or publication ethics. (Please upload your review as an attachment if it exceeds 20,000 characters)

Reviewer #1 Comment #1: Thank you for the opportunity to review this manuscript on ChatGPT and similar AI tools. As an academic, the topic holds great relevance and has been widely discussed. However, I believe that a comprehensive review of existing literature is essential for advancing knowledge in any field. Literature reviews serve as the foundation for new research, identifying gaps, building upon previous studies, and contributing to the overall body of knowledge. Unfortunately, this manuscript suggests that the current literature is insufficient in providing a comprehensive understanding of the subject matter.
In some cases, the insufficiency of literature can be attributed to the field being relatively new or rapidly evolving. New developments and paradigms take time to be reflected in the literature, resulting in limited studies, replication, and consensus on key concepts or theories. This dynamic nature poses challenges for researchers attempting to establish a comprehensive literature base. Recognizing the insufficiency of existing literature is crucial for addressing gaps and advancing knowledge in the field. However, this manuscript falls short in providing a full and comprehensive picture of how ChatGPT can be utilized in broader contexts, such as education, healthcare, and research. The literature on these specific applications was limited to one paragraph each, which is insufficient.

Reviewer #1 Response #1: We thank the reviewer for the comments and agree that this is a rapidly developing field. We have expanded upon our introduction and background in all three realms of education, healthcare, and research. We have furthermore linked to more comprehensive reviews for interested readers for detailed discussion and literature review beyond the scope of our paper.

Reviewer #1 Comment #2: Additionally, the manuscript heavily relies on unnecessary graphics and images, which could have been condensed to reduce fluff. The demographic information could have been presented more concisely. Furthermore, the discussion section lacks a clear data interpretation process and a deeper understanding of the results. It appears that the researchers randomly selected comments without providing a comprehensive analysis.

Reviewer #1 Response #2: We have condensed some of our figures and presented our demographic information in Supplemental Table 1 in a concise format. We included all comments raised during the Question and Answer session and added more comprehensive analysis.

Reviewer #1 Comment #3: Moreover, there were frequent APA errors throughout the manuscript, which need to be addressed.

Reviewer #1 Response #3: We have adhered to the Vancouver style as outlined by PLOS. We are happy to work on any additional formatting requests from a copy editor.

Overall, I appreciate the opportunity to review this manuscript. However, improvements are needed to provide a more comprehensive literature review, condense unnecessary elements, and address the APA errors.

Reviewer #2 Comments: The paper is not as per journal format.
The font sizes are different in several placesInstead of bibliography it should be referencesIf possible Need to cite more papers related to chatGPT and related to this research
Figures titles are not as per journal standards and make sure to use any templates related to plus one available

Reviewer #2 Responses: We have corrected these stylistic errors, cited more papers related to the research, and corrected figure titles and references. We are happy to consider additional formatting changes as suggested by a copy editor.

6. PLOS authors have the option to publish the peer review history of their article ([what does this mean?](https://journals.plos.org/plosone/s/editorial-and-peer-review-process#loc-peer-review-history)). If published, this will include your full peer review and any attached files.


If you choose “no”, your identity will remain anonymous but your review may still be made public.


**Do you want your identity to be public for this peer review?** For information about this choice, including consent withdrawal, please see our [Privacy Policy](https://www.plos.org/privacy-policy).

Reviewer #1: **Yes:** ASHLEY GREENE

Reviewer #2: **Yes:** Dinesh Kalla
