# Supplementary material for: An exploratory survey about using ChatGPT in education, healthcare, and research
Source: PLoS One. 2023 Oct 5;18(10):e0292216. doi: 10.1371/journal.pone.0292216 (PMC10553335; doi:10.1371/journal.pone.0292216)
Supplement: Attachment — Submitted filename: Response to reviewer comments.docx [file pone.0292216.s003.docx]

Dear Dr. Clark and the *PLOS One* editorial team,

Thank you for your comments. We have revised our manuscript and included a point-by-point response to the reviewer comments below.

Reviewer #1: Thank you for your revisions. At this time, I feel my original comments were adequately addressed. I would recommend a copy editor to do the final polishing of the manuscript, and to help create APA style tables. Currently Table 1 takes 3.5 pages and could be broken down into several smaller table to help readability.

Thanks for your suggestion. We have broken down Table 1 into two smaller tables and applied the APA style. We also had the paper reviewed and improved for readability.

Reviewer #2: All comments have being addressed and this paper can be accepted with no further changes.

Thanks
