# Supplementary material for: An exploratory survey about using ChatGPT in education, healthcare, and research
Source: PLoS One. 2023 Oct 5;18(10):e0292216. doi: 10.1371/journal.pone.0292216 (PMC10553335; doi:10.1371/journal.pone.0292216)
Supplement: Attachment — Submitted filename: Response to reviewer comments.docx [file pone.0292216.s004.docx]

Dear Dr. Clark,

Thank you so much for your comments and excellent suggestions. Please accept our apologies for minor formatting and grammar mistakes.

We have reread and revised our manuscript with minor changes. We accepted all your suggestions and include a point-by-point response below.

Fun paper and thanks for all of the revisions. I have a few more--must have give feedback to tooooo many doc students today. I look forward to these very minor corrections and the publication of your paper.

Abstract---Materials and Methods
Please add the word ‘a’. before Fisher’s Exact
Done

Page 4---5th line. : embedded in used educational resources
Improved

Again page 4 first paragraph
Increased likelihood of plagiarism, propagation of irrelevant or inaccurate
information in student essays, and challenges of assessing students in the presence of
technologies such as ChatGPT are highlighted [10].
Improved

Please rephrase:
Using ChatGPT increases the likelihood of plagiarism, the propagation of irrelevant or inaccurate information in students’ essays, and challenges in assessing students’ work when technologies, such as ChatGPT, are available.
Improved

Page 5 Methods
4th line --- add ‘the’ before 27th of January 2023
Done

Then last two lines The Northwestern IRB granted ‘an’ exemption
Done

Notice that you are missing some articles. I have become extremely sensitive to this issue because articles are not used in ASL and my grant office gave me what for. So if this continues I will have to ask you to get a professional to check.
We have reread and improved the whole paper and paid specific attention to articles

Page 6 8th line----platform, they are were consenting
Done

Then under Survey Results
The smallest group was were medical trainees
Done

Next line
Respondents), and ‘the’ second smallest by was
Done

Page 7
Compared to 15.9% who had no interest in using it (p<0.001; Fig.1)
Done

Page 11Possible Negative impacts
Second line
Improved

About used sources ‘used’

Page 12 last line
Such as ‘the’ Google search engine

Done
